# Congenital Anomalies and Termination of Pregnancy in Iran

**DOI:** 10.1155/2012/574513

**Published:** 2012-04-24

**Authors:** Bahram Samadirad, Zhila Khamnian, Mohammad Bager Hosseini, Saeed Dastgiri

**Affiliations:** ^1^Research Centre of Iranian Legal Medicine Organization, 5166615739 Tabriz, Iran; ^2^Department of Community Medicine, National Public Health Management Centre (NPMC), School of Medicine, Tabriz University of Medical Sciences, 5166615739 Tabriz, Iran; ^3^Department of Neonatology, Pediatric Health Research Centre, Tabriz University of Medical Sciences, 5166615739 Tabriz, Iran

## Abstract

The aim of this study was to document some epidemiological features of termination of pregnancy for birth defects in Iran. We studied 603 pregnant women who were diagnosed/recommended for the termination of pregnancy as having a fetus with some types of birth defect(s). Most women (87.2 percent) had at least one ultrasound examination. The proportion of other screening tests including amniocentesis and genetic tests were 2.8 and 4.6 percent, respectively. Of 603 women, 201 terminated the pregnancy giving a prevalence rate of 33.3 percent (CI 95%: 29.6–37.6). The remaining 402 subjects were unable to get the permission for abortion because of untimely diagnosis/application for termination (20th week of pregnancy and/or later). Forty-eight percent of termination of pregnancies was performed before the 18th week of pregnancy. Neural tube defects, limb deformation, hydrops fetalis, hydrocephaly, and chromosomal anomalies including Down syndrome accounted proportionally for about 65 percent of defects eligible for abortion in the region. Although the rate of termination of pregnancy for birth defects is acceptable at the current situation in the country, more efforts should still be made to convince the community authorities to give more possibility and ease for the termination of pregnancy for congenital anomalies.

## 1. Introduction

Therapeutic abortion is defined as intentional termination of pregnancy performed or authorized by a physician in order to save the mother's life and health. Termination of pregnancy is legally allowed in Iran if three specialist physicians confirm that the outcome of pregnancy may be harmful, for any reason, for mother/family during pregnancy or after birth. The reasons for this termination include the following circumstances: (a) complications during the pregnancy endangering mother's health and (b) termination of pregnancy due to major malformation of fetus [1, 2].

According to the current laws in Iran, the permission for termination is only issued before the 20th week of pregnancy. According to the country's current religious rules and traditions, it is believed that fetal viability occurrs after the 20th week, and it would not then be allowed to abort the fetus after this time [3, 4].

Currently, in Iran, one of the main reasons for termination of pregnancy is congenital anomalies. Many of the serious maternal and fetal conditions are now easily permitted for abortion in the country (Tables 3 and 4) [2, 4]. In addition to those conditions, if three specialist physicians confirm that the outcome of pregnancy may be harmful for any other reason to mother/family during pregnancy or after birth, the abortion will also be permitted even if it is not included in the list.

The aim of this study was to describe the epidemiological profile of induced abortions in women with birth defects in the northwest of Iran during 2010.

## 2. Methods

Tabriz Registry of Congenital Anomalies (TRoCA) covers an annual average of 20000 births in the northwest of Iran, including live births and stillbirths [5]. As for current screening programme and pregnancy checkups in Iran, all pregnant women are routinely examined in public or private clinics by a gynecologist, obstetrician, or midwife. Ultrasound examination is one of the routine diagnostic tests for maternal care in the country. It is usually performed 3-4 times during pregnancy period beginning from early weeks and then in the 12th, 22nd, and 32nd weeks of pregnancy. It is recommended to do one of the ultrasound examinations between 14th and 16th weeks for birth defect(s) diagnosis. The same routine care are given after birth for infants by neonatologist or pediatrician—both at birth and at hospital discharge—for possible intervention and treatment. The examinations include assessment of maternal health status, maturity, and congenital anomalies of fetus/infant during pregnancy and after birth. We studied 603 women with a pregnancy complicated by a birth defect in the population under the TRoCA programme. They were identified by medical diagnostic tests during the antenatal period as having a fetus with birth defect(s). Pregnant mothers were then referred to three specialist physicians for final confirmation of congenital anomalies. Of them, 201 mothers were legally allowed to proceed to abortion (Group I), and the remaining 402 subjects were unable to obtain permission for abortion (Group II). The definition of the congenital anomalies for the purposes of this study is based on the standard coding system of the International Classification of Diseases (ICD) and British Paediatric Association (BPA). Thus, the study subjects comprised all pregnant women who were diagnosed/recommended for the termination of pregnancy as having a fetus with some types of birth defect(s) with a primary diagnosis under one of the anomalies headings.

After obtaining an informed consent from the participants, an expert physician performed the clinical examinations and took the medical history of the subjects. All mothers were also asked to complete a brief questionnaire for demographic characteristics and socioeconomic status.

Approval for this study was obtained from Regional Committee of Medical Ethics of Tabriz University of Medical Sciences.

Descriptive statistics (including proportions, means, and standard deviations) and 95% confidence intervals were used for statistical analysis of data. 

## 3. Results

There were a total of 22524 births (22320 live and 204 stillbirths) in the area in 2010. Of those, 639 pregnancies (2.8 percent, CI95%: 2.6–3.0) were identified as having a fetus with birth defect(s). Demographic characteristics of the study subjects are shown in Table 1. The mean age of mothers was 28.3 years (range: 14–44 years). Thirty-five percent of subjects had consanguineous marriage. Eighty cases had a history of anomaly in previous pregnancies, 65 had the same anomaly in father's family compared to the 42 cases with the similar anomaly in mother's family. Most women (87.2 percent) had at least one ultrasound examination. The proportions of other screening tests including amniocentesis and genetic tests were 2.8 and 4.6 percent, respectively. Of 603 women diagnosed as having a baby with birth defect, 201 terminated the pregnancy giving a prevalence rate of 33.3 percent (CI95%: 29.6–37.6). Forty-eight percent of termination of pregnancies was performed before the 18th week of pregnancy. Neural tube defects, limb deformation, hydrops fetalis, hydrocephaly, and chromosomal anomalies including Down syndrome accounted proportionally for about 65 percent of defects eligible for abortion in the region (Table 2).

## 4. Discussion

This study was carried out to produce the epidemiological profile of induced abortions for birth defects in Iran. The prevalence of pregnancies complicated by congenital anomalies was estimated 2.8 percent of total births in the region. One-third of women with a fetus with birth defect(s) had consanguineous marriage. Most of them reported to have at least one ultrasound examination during the pregnancy. Major anomalies for abortion included neural tube defects, limb deformation hydrops fetalis, hydrocephaly, and chromosomal anomalies. Our findings were consistent with other studies previously reported in terms of demographic and socioeconomic indicators [6–9]. However, one of the main reasons for the termination of pregnancies was thalassemia in previous studies where it was rare in our investigation. This shows that thalassemia is now under the control of health care system in the country as reported before [10].

In three out of every five pregnancies with congenital abnormalities, consent for terminating the pregnancy could not be obtained as the diagnosis/application only occurred after 20 weeks indicating that early diagnosis and timely request would be essential to get the abortion permit.

It is concluded that one in almost three pregnancies prenatally diagnosed with birth defect(s) is now legally terminated in Iran. Although this figure is acceptable at the current situation in the country, more efforts should still be made to (a) convince the community authorities to give more possibility and ease for the termination of pregnancy for congenital anomalies, (b) improve the diagnostic facilities for early and timely diagnosis of fetal anomalies, (c) train medical staff in the clinical indications of therapeutic abortion of birth defects, and (d) provide the necessary information to young couples about the suitable age of pregnancy and about folic acid consumption before and during pregnancy.

## Figures and Tables

**Table 1:** Basic characteristics of study subjects.

		Group I	*n* = 201	95% CI		Group II	*n* = 402	95% CI	
		Number	Percent	Lower	Upper	Number	Percent	Lower	Upper
Mother literacy	Primary/secondary	120	59.7	52.7	66.7	275	68.4	63.7	72.9
	High school	60	29.9	23.9	36.3	98	24.4	20.4	28.6
	University	21	10.4	6.5	14.9	29	7.2	4.7	9.7

Father literacy	Primary/secondary	134	66.7	60.7	73.6	253	62.9	58.0	67.9
	High school	42	20.9	14.9	26.9	89	22.1	17.9	26.1
	University	25	12.4	8.0	17.4	60	14.9	11.7	18.4

Mother job	Housewife	181	90.0	86.1	94.0	370	92.0	89.3	94.5
	Employed	20	10.0	6.0	13.9	32	8.0	5.2	10.7

Family income (per month, US $)	Less than 400	129	64.2	57.7	70.1	245	60.9	56.2	65.7
	400–800	56	27.9	21.4	34.3	139	34.6	29.6	39.3
	More than 800	16	7.9	4.5	11.9	18	4.5	2.7	6.5

Type of marriage	Familial	56	27.9	21.9	33.8	154	38.3	33.6	43.5
	Nonfamilial	145	72.1	66.2	78.1	248	61.7	56.5	66.4

		Mean		(95% CI)		Mean		(95% CI)	

Mother age		29.14		(28.24–29.97)		27.87		(27.34–28.41)	
Father age		33.47		(32.51–34.42)		33.61		(33.04–34.17)	

**Table 2:** Termination of pregnancy by the type of birth defects.

Type of anomaly	Group I		Group II		Total	
	Number	Percent	Number	Percent	Number	Percent
Down syndrome	14	7.0	37	9.2	51	8.5
Hydrocephaly	19	9.5	50	12.4	69	11.4
Chromosomal anomaly	3	1.5	9	2.2	12	2.0
Limb deformation	14	7.0	48	11.9	62	10.3
Heart anomaly	7	3.5	5	1.2	12	2.0
Hydrops fetalis	26	12.9	35	8.7	61	10.1
Spinal muscular atrophy	8	4.0	7	1.7	15	2.5
Oligohydramnios	21	10.4	28	7.0	49	8.1
Microcephaly	7	3.5	25	6.2	32	5.3
Polyhydramnios	8	4.0	19	4.7	27	4.5
Major thalassemia	6	3.0	0	0.0	6	1.0
Neural tube defects	44	21.9	92	22.9	136	22.6
Others	24	11.9	47	11.7	71	11.8

**Table 3:** List of maternal indications of therapeutic abortion in Iran.

(1)	Any of the cardiac valve diseases with functional class 3-4 heart failure that is not reversible to class 2
(2)	Any kind of acute heart disease except coronary heart disease that has reached functional class 3-4 (e.g., myocarditis and pericarditis)
(3)	History of dilated cardiomyopathy in previous pregnancies
(4)	Marfan syndrome, when ascending aorta is wider than 5 cm
(5)	Eisenmenger's syndrome
(6)	Pregnancy-induced fatty liver
(7)	Grade 3 esophageal varicosities
(8)	History of esophageal varicosities hemorrhage followed by portal hypertension
(9)	Uncontrollable autoimmune hepatitis
(10)	Renal failure
(11)	Hypertension which is not controllable with permitted drugs during pregnancy
(12)	Any of the pulmonary diseases that lead to pulmonary hypertension even to a mild degree (emphysema, fibrosis, diffuse bronchiectasis)
(13)	Any infection with HIV virus which has entered into the phase of AIDS disease
(14)	Hypercoagulability when using heparin leads to progression of other diseases that can threat's motheren life
(15)	Active uncontrollable SLE which has involved a major organ
(16)	Vasculitis (when major organs are involved)
(17)	All CNS masses with considering their type and location, when beginning the treatment is dangerous to fetus and without treatment mother-life may be threatened
(18)	Pemphigus vulgaris and severe generalized psoriasis and advanced melanoma
(19)	Multidrug-resistant epilepsies
(20)	MS cases in which the patient is disabled
(21)	Myasthenia gravis
(22)	Some type of motor neuron diseases like ALS which is intensified following pregnancy and will seriously endanger mother's life

**Table 4:** List of fetal indications of therapeutic abortion in Iran.

(1)	Osteogenesis imperfecta
(2)	Osteochondrodysplasia
(3)	Osteopetrosis and infantile neuroaxonal dystrophy
(4)	Bilateral renal agenesis
(5)	Polycystic kidney
(6)	Multicystic dysplastic kidney
(7)	Potter syndrome
(8)	Congenital nephrotic syndrome and hydrops
(9)	Severe bilateral hydronephrosis
(10)	Alpha thalassemia and hydrops fetalis
(11)	Thrombotic disorders
(12)	Trisomy 13, 18, 3, 16, and 8
(13)	Anencephaly
(14)	Cat cry syndrome
(15)	Holoprosencephaly
(16)	Syringomyelia
(17)	Cranioschisis
(18)	Meningoencephalocele
(19)	Meningohydroencephalocele
(20)	Thanatophoric dysplasia
(21)	Cyclopia with holoprosencephaly
(22)	Ichthyosis congenita
(23)	Schizencephaly

## Appendix

See Tables 3 and 4.

## References

[1] Erfani A, McQuillan K (2008). Rates of induced abortion in Iran: the roles of contraceptive use and religiosity. *Studies in Family Planning*.

[2] (1991 ). Essentials of islamic judiciary laws. *Iran News Paper*.

[3] Sadr S (2002). *Indications for Abortion in Iran*.

[4] Hedayat KM, Shooshtarizadeh P, Raza M (2006). Therapeutic abortion in Islam: contemporary views of Muslim Shiite scholars and effect of recent Iranian legislation. *Journal of Medical Ethics*.

[5] TRoCA http://troca.tbzmed.ac.ir.

[6] Nojomi M, Akbarian A (2006). Burden of abortion: induced and spontaneous. *Archives of Iranian Medicine*.

[7] Liu S, Joseph KS, Wen SW (2002). Trends in fetal and infant deaths caused by congenital anomalies. *Seminars in Perinatology*.

[8] Bazmi S, Behnoush B, Kiani M, Bazmi E (2008). Comparative study of therapeutic abortion permissions in central clinical department of Tehran Legal Medicine Organization before and after approval of law on abortion in Iran. *Iranian Journal of Pediatrics*.

[9] Ghadipasha M, Aminian Z (2007). The study of abortion licenses issued by legal medicine office in kerman. *Journal of Kerman University of Medical Sciences*.

[10] Aghajani H, Samavat A, Haghazali M, Valizadeh F, Sarbazi G (2009). Primary health care: an approach to community control of genetic and congenital disorders. *Iranian Journal of Public Health*.

